# Relationship between tooth loss and sarcopenia in suburban community-dwelling older adults in Shanghai and Tianjin of China

**DOI:** 10.1038/s41598-022-11714-7

**Published:** 2022-05-10

**Authors:** Feng Wang, Jingru Wang, Peipei Han, Yuewen Liu, Weibo Ma, Hui Zhang, Ning Wu, Sijia Sang, Yining Xia, Jiangtao Pan, Yang Liu, Fandi Xie, Shumeng Niu, Hao Hu, Hongbing Wang, Ying Yu, Qi Guo

**Affiliations:** 1 Department of Rehabilitation Medicine, Shanghai University of Medicine and Health Sciences Affiliated Zhoupu Hospital, 1500 Zhouyuan Road, Pudong New District, Shanghai, 201318 China; 2 Shanghai University of Medicine and Health Sciences, 279 Zhouzhu Highway, Shanghai, 201318 China; 3 Shanghai University of Traditional Chinese Medicine, Shanghai, China; 4 Shanghai Hongkou District Jiangwan Hospital, Shanghai, China; 5 Department of Rehabilitation, Shanghai Fourth Rehabilitation Hospital, Shanghai, China

**Keywords:** Risk factors, Oral diseases, Epidemiology

## Abstract

Both sarcopenia and loss of teeth are associated with aging. The purpose of this study was to investigate potential relationships between tooth loss and sarcopenia and its components in suburban community-dwelling older adults of Shanghai and Tianjin, China. The subjects were 1494 people over 60 years of age (40.7% men; aged 71.64 ± 5.97 years) from Chongming District of Shanghai and Hangu District of Tianjin. Asian Working Group for Sarcopenia(AWGS) criteria were used to define sarcopenia. Muscle mass, muscle strength, and physical performance were assessed using a bioelectrical impedance analyzer, a grip strength test, and a four-meter walk test, respectively. The subjects were divided into groups depending on self-reported loss of teeth. Our studies found no correlation between tooth loss and sarcopenia or muscle mass. However, the walking speed of female participants with at least 10 teeth lost was 0.059 m/s slower than that of participants with fewer than 10 teeth lost (*p* < 0.001), and grip strength was 1.577 kg lower among male participants with at least 10 teeth lost than among males with fewer than 10 teeth lost (*p* = 0.023). These results are consistent with the importance of good oral hygiene in preventing declines of physical performance in older adults.

## Introduction

China's aging problem is becoming increasingly serious, with 164.5 million people aged 65 and above^1^. At the same time, incidence of age-related sarcopenia is also on the rise, and it has become an important focus of geriatric research^2^. Sarcopenia is defined as the age-related loss of indicators of skeletal muscle quality, including muscle mass, strength and physical performance^3^. In older adults, sarcopenia is associated with an increase in adverse outcomes, including falls, functional decline, weakness and death^4,5^. Therefore, active prevention and treatment of sarcopenia is particularly important.

Older adults also face increasingly serious problems with oral health associated with declines of physical and mental health^6^. Oral health problems can have a significant impact on the elderly, leading to discomfort, pain and tooth loss. Tooth loss, in particular, is an indicator of poor oral health^7^. Periodontitis is the main cause of tooth loss, and it closely related to the increase of the pro-inflammatory factors interleukin-1 (IL-1), tumor necrosis factor-α (TNF-α) and IL-6^8^. In addition, the local inflammation caused by poor oral health can induce a systemic inflammatory response, these systemic changes, such as the accumulation of pro-inflammatory cytokines, also occur in muscle injury^9^. The inflammation associated with poor oral health may, then, affect physical fitness, especially muscle mass, muscle strength and muscle function^9^. In addition, adult tooth loss may affect physical fitness and contribute to sarcopenia by reducing diet quality and the intake of nutrients^10^. Therefore, it is reasonable to think that tooth loss is causally related to sarcopenia and its components.

In a recent study, poor oral health, including tooth loss, was shown to be a predictor of future sarcopenia in community-dwelling older adults^11^, while two other studies identified tooth loss as a risk factor for sarcopenia in adults^12,13^. Despite the significant correlations that have been identified, there are relatively few studies on tooth loss and sarcopenia, and those that have been performed typically did not evaluate the use of denture, which has been shown to impact nutritional status^14^. Therefore, further research is necessary in order to establish whether tooth loss is a risk factor for sarcopenia in the elderly. In addition, the relationship between tooth loss and muscle mass, strength and function, which are indicators of sarcopenia, remains controversial^15–20^. There are relationships of tooth loss relative to the specific components of sarcopenia, including muscle mass, muscle strength, and physical performance.

Here, we conducted a cross-sectional study to determine the relationship between tooth loss and sarcopenia and its contributing factors among suburban community-dwelling older adults in Shanghai and Tianjin of China, so as to provide a reference for predicting decreased physical performance and identifying high-risk groups. Given the ease with which oral health interventions can be integrated into daily life, it is important to determine whether such interventions can be used to reduce age-related risks of sarcopenia and decline of physical performance.

## Results

The data of 1614 participants from Hangu District, Tianjin and Chongming District, Shanghai were collected in August 2018 and June to July 2019. A total of 1494 participants were included for analysis in this study. Tables 1 and 2 show the socioeconomic characteristics, demographics and disease statuses of the participants. Of the participants, 609 (40.7%) were male. It was found that age, BMI, relative skeletal muscle mass index, grip strength, average walking speed, overall sarcopenia status, and denture status were significantly different between the groups that were categorized according to the number of teeth lost (Tables 1, 2).

**Table 1:** Characteristics of male study group, stratified by number of tooth loss.

Variable	Number of tooth loss		*p*-value
	0 to 9 (n = 335)	≥ 10 (n = 274)	
Age (y)	70.07 ± 5.26	74.23 ± 6.00	< 0.001
BMI	24.25 ± 3.03	22.93 ± 3.54	< 0.001
ASM/Ht^2^ (kg/m^2^)	7.86 ± 0.97	7.51 ± 0.79	< 0.001
Grip strength (kg)	31.13 ± 7.44	27.57 ± 7.78	< 0.001
Walk speed (m/s)	1.14 ± 0.24	1.07 ± 0.25	0.001
IPAQ (Met/week)	4164 (1533, 8400)	3786 (1626, 6426)	0.033
Widowed, n (%)	25 (7.5)	33 (12.0)	0.057
Living alone, n (%)	36 (10.7)	33 (12.0)	0.615
Illiteracy	22 (6.6)	27 (9.9)	0.251
**Monthly income, n (%)**			
< 157.2 USD	49 (14.7)	40 (14.7)	0.363
**Smoking, n (%)**			
Current smokers	92 (27.5)	111 (40.7)	< 0.001
**Drinking, n (%)**			
Daily drinkers	78 (23.6)	78 (29.0)	0.003
**Nutritional status**			
Well-nourished	272 (81.2)	195 (71.2)	0.014
At risk of malnutrition	61 (18.2)	76 (27.7)	
Malnourished	2 (0.6)	3 (1.1)	
**Disease, n (%)**			
Hypertension	223 (66.6)	171 (62.4)	0.285
Hypercholesterolemia	204 (60.9)	139 (50.7)	0.012
Diabetes	54 (16.1)	53 (19.3)	0.298
Stoke	26 (7.8)	22 (8.1)	0.897
Sarcopenia	19 (5.7)	40 (14.6)	< 0.001
Depression	32 (9.8)	27 (9.9)	0.954
Cognitive impairment	38 (11.8)	40 (15.2)	0.241
**Brushing frequency, n (%)**			
≥ 2 times/day	113 (33.7)	78 (28.4)	0.161
< 2 times/day	222 (66.3)	196 (71.6)	
**Brushing time, n (%)**			
< 2 min	181 (53.9)	153 (56.0)	0.616
≥ 2 min	154 (46.1)	121 (44.0)	
Gingival bleeding, n (%)	44 (13.2)	14 (5.2)	0.001
Denture status, n (%)	86 (25.7)	184 (67.2)	< 0.001

Data are shown as mean ± SD or number (percentage).

*BMI* body mass index, *ASM/Ht*^2^ ASM, appendicular lean mass, *Ht* Height, *IPAQ* International Physical Activity Questionnaire, *MET/week* metabolic equivalent task minutes per week.

**Table 2:** Characteristics of female study group, stratified by number of tooth loss.

Variable	Number of tooth loss		*p*-value
	0 to 9 (n = 436)	≥ 10 (n = 449)	
Age (y)	69.42 ± 5.19	73.40 ± 6.03	< 0.001
BMI	24.73 ± 3.62	23.93 ± 3.47	0.001
ASM/Ht^2^ (kg/m^2^)	6.36 ± 0.94	6.08 ± 0.79	< 0.001
Grip strength (kg)	19.09 ± 5.54	17.46 ± 5.56	< 0.001
Walk speed (m/s)	1.08 ± 0.17	0.99 ± 0.23	< 0.001
IPAQ (Met/week)	4519 (2373, 8363)	4110 (1688, 8078)	0.643
Widowed, n (%)	91 (20.9)	142 (31.6)	0.001
Living alone, n (%)	60 (13.8)	101 (22.5)	0.001
Illiteracy	106 (24.4)	110 (24.5)	0.691
**Monthly income, n (%)**			
< 157.2 USD	93 (21.3)	97 (21.7)	0.956
**Smoking, n (%)**			
Current smokers	27 (6.2)	37 (8.3)	0.433
**Drinking, n (%)**			
Daily drinkers	13 (3.1)	24 (5.4)	0.104
**Nutritional status**			
Well-nourished	344 (78.8)	340 (75.8)	0.288
At risk of malnutrition	91 (21.0)	105 (23.3)	
Malnourished	1 (0.2)	4 (0.9)	
**Disease, n (%)**			
Hypertension	279 (64.0)	316 (70.4)	0.043
Hypercholesterolemia	302 (69.3)	319 (71.0)	0.563
Diabetes	90 (20.7)	84 (18.8)	0.479
Stoke	20 (4.7)	25 (5.6)	0.528
Sarcopenia	43 (9.9)	80 (17.8)	0.001
Depression	54 (12.6)	57 (12.9)	0.881
Cognitive impairment	79 (19.3)	103 (24.5)	0.067
**Brushing frequency, n (%)**			
≥ 2 times/day	192 (44.0)	200 (44.6)	0.862
< 2 times/day	244 (56.0)	249 (55.4)	
**Brushing time, n (%)**			
< 2 min	268 (61.5)	259 (57.7)	0.255
≥ 2 min	168 (38.5)	190 (42.3)	
Gingival bleeding, n (%)	70 (19.6)	45 (10.5)	< 0.001
Denture status, n (%)	154 (35.3)	333 (74.2)	< 0.001

Data are shown as mean ± SD or number (percentage).

*BMI* body mass index, *ASM/Ht*^2^ ASM, appendicular lean mass, *Ht* Height, *IPAQ* International Physical Activity Questionnaire, *MET/week* metabolic equivalent task minutes per week;

A binary logistic regression model was applied, and the data were adjusted for the covariates age, BMI, district, IPAQ, monthly income, education, smoking, drinking, denture status, depression, nutrition status, hypertension, and diabetes. We did not identify a statistical relationship between tooth loss and sarcopenia (Table 3). Next, a multivariate linear regression model was developed, after adjusting for the potentially confounding factors. In this analysis, muscle mass was not associated with tooth loss in either sex group. In men, grip strength was 1.577 kg lower in those with at least 10 teeth lost as compared with those with nine or fewer teeth lost (*p* = 0.023). Among women, participants who had lost at least 10 teeth had an average walking speed that was 0.059 m/s slower than that of participants with nine or fewer teeth lost (*p* < 0.001) (Table 4).

**Table 3:** Binary logistic regression of sarcopenia, showing effects as odds ratios and 95% confidence intervals.

Independent variable	Model 1		Model 2		Model 3	
	OR (95%CI)	*p*-value	OR (95%CI)	*p*-value	OR (95%CI)	*p*-value
**Male**						
0 to 9	Ref.		Ref.		Ref.	
≥ 10	1.369 (0.703–2.665)	0.356	1.633 (0.751–3.547)	0.216	1.585 (0.696–3.231)	0.251
**Female**						
0 to 9	Ref.		Ref.		Ref.	
≥ 10	1.368 (0.843–2.218)	0.205	1.242 (0.727–2.123)	0.428	1.261 (0.727–2.188)	0.409

Model 1: adjusted for age, BMI, district. Model 2: adjusted for age, BMI, district, IPAQ, monthly income, education, smoking, drinking, denture status. Model 3: adjusted for age, BMI, district, IPAQ, monthly income, education,smoking, drinking, denture status, depression, nutrition status, hypertension, diabetes.

*OR* odds ratio, *CI* confidence interval.

**Table 4:** Linear association between the number of tooth loss and ASM/Ht^2^, grip strength, walk speed.

Independent variable	Model 1		Model 2		Model 3	
	β	*p*-value	β	*p*-value	β	*p*-value
**ASM/(Ht)**^2^** (kg/m**^2^**)**						
Male						
0 to 9	Ref.		Ref.		Ref.	
≥ 10	− 0.066	0.289	− 0.100	0.150	− 0.083	0.212
Female						
0 to 9	Ref.		Ref.		Ref.	
≥ 10	− 0.044	0.364	− 0.011	0.830	− 0.013	0.810
**Grip strength (kg)**						
Male						
0 to 9	Ref.		Ref.		Ref.	
≥ 10	− 1.254	0.043	− 1.707	0.013	− 1.577	0.023
Female						
0 to 9	Ref.		Ref.		Ref.	
≥ 10	− 0.614	0.112	0.797	0.055	− 0.730	0.083
**Walk speed (m/s)**						
Male						
0 to 9	Ref.		Ref.		Ref.	
≥ 10	− 0.029	0.149	− 0.041	0.063	− 0.036	0.107
Female						
0 to 9	Ref.		Ref.		Ref.	
≥ 10	− 0.044	0.001	− 0.063	< 0.001	− 0.059	< 0.001

Model 1: adjusted for age, BMI, district. Model 2: adjusted for age, BMI, district, IPAQ, monthly income, education, smoking, drinking, denture status. Model 3: adjusted for age, BMI, district, IPAQ, monthly income, education, smoking, drinking, denture status, depression, nutrition status, hypertension, diabetes.

## Discussion

The results of this study suggest that among the elderly living in suburban communities of coastal cities in China, the self-reported tooth loss of the elderly has a significant statistical relationship with male muscle strength and female average walking speed. Therefore, dental examination and dental nursing guidance are essential in a more comprehensive assessment of physical performance of the elderly. Considering that the association between the number of teeth and physical performance remains controversial, the results of this study have certain reference value.

### The relationship between tooth loss and sarcopenia

Our study disagrees with previous research that has linked general sarcopenia to oral health. In one recent study, subjects with fewer than 20 natural teeth had an adjusted odds ratio for sarcopenia of 1.96 for men and 2.86 for women, compared with those with full dentition^12^. Another cross-sectional study also showed that impaired dentition was significantly associated with sarcopenia in Japanese adults living in the community^13^. In a meta-analysis of 27 studies reviewed, a possible association between oral health status and sarcopenia was found; however, most of the studies included in this analysis were cross-sectional studies in Japan, and the results therefore are not representative^21^.

On the other hand, after adjusting for covariate factors, we found no association between tooth loss and sarcopenia in either sex. One possible explanation for the disagreement may result from the varying ages of study participants. Age is an important factor in sarcopenia: physical function declines by approximately 4% every year after the age of 65, and movement disorders become more common, affecting 24% of adults over 75 years old^22,23^. We used relatively young older adults, and the age range of the population included in our study was fairly small; male and female study participants were aged, on average, 71.94 ± 5.97 years and 71.44 ± 5.97 years, respectively. In other studies, either the ages of participants exceeded 75 years or the range of ages of inclusion was too wide to permit generalizable claims^12,13^. These differences in participant ages, then, may explain why other groups found relationships between tooth loss and sarcopenia, even though no such relationships seem to exist in age-matched groups. Notably, thus far, associations between tooth loss and sarcopenia have been reported only rarely, and no firm conclusions have been drawn. Further large longitudinal studies are needed to test these proposed relationships.

### The relationship between tooth loss and muscle mass

While connections between sarcopenia in general and tooth loss are controversial, more detailed studies have suggested that links between tooth loss and one component of sarcopenia, low muscle mass, may occur through inflammatory and nutritional pathways^24,25^. For example, one key factor in tooth loss, periodontitis, increases levels of IL-6 and TNF-α in gingival tissue^26^. Resultant systemic increases of these inflammatory cytokines can cause loss of muscle mass^27,28^. In support of this mechanistic connection, Akinari et al. found that tooth loss was an independent risk factor for decreased muscle mass in men but not in women^20^. Notably, there are limitations to this study, especially regarding the relatively small sample size.

In a recent longitudinal study, poor oral health, including tooth loss, was shown to be a predictor of future muscle loss in older people living in the community^11^. A Korean study similarly showed that participants with at least 20 remaining natural teeth had more muscle mass on average than those with fewer than 20 remaining natural teeth. While this latter study ostensibly demonstrated a connection in men and women, after correcting for confounding factors, the number of remaining teeth and muscle mass were found to correlate in men but not in women over age 65^29^. It is speculated that the reason for gender differences in this correlation may be that men and women tend to make different food choices, with women tending to eat more fruits and vegetables than men. This lifestyle connection may lead to a limiting of protein intake in women and thus gender-related differences in muscle mass dynamics.

Our findings are inconsistent with several previous studies regarding muscle mass, however. Although we did identify differences in muscle mass between men and women, after adjusting for confounders, we found no correlation between tooth loss and muscle mass. One reason for these differences may involve the assessment tool. We used a reliable method that is specified by Asian Working Group for Sarcopenia^30^, and this method would be expected to lead to highly accurate results. In addition, our study population lives in the suburban community, and many of the participants were of low economic status and had been engaged in agricultural labor during their working years^31^. In China, rural residents are currently more physically active than their urban counterparts^32^. Studies have shown that physical activity have anti-inflammatory effects^33^. Inflammatory cytokines have been proved to promote muscle atrophy, and finally stimulate protein catabolism and inhibit muscle synthesis^34^. Participation in physical activity can reduce the levels of inflammatory markers C-reactive protein, tumor necrosis factor and interleukin. These markers have been found to play an important role in the pathogenesis of oral diseases^35^. The present study also shows that participation in physical activity is favourably associated with some self-reported oral health correlates^33^.

### The relationship between tooth loss and grip strength

Grip strength is a commonly used indicator of total body strength and is a known predictor of disability and mortality^36^. A recent Chinese study showed no link between tooth loss and grip strength in people over the age of 60^37^. In a Japanese community study involving participants ages 40 to 70, there was also no correlation found between the number of teeth and grip strength in either sex^20^. The results of this latter study are limited because of the lack of consideration of confounding factors, including socioeconomic status and the use of dentures, both of which have clear connections to tooth loss. In particular, the use of dentures can improve the chewing ability of older adults and thus can improve nutrient intake and nutritional status, which would affect physical performance and muscle strength^38^. Recently, Bramantoro et al.^9^ pointed out that poor oral health has a negative impact on cognitive function and physical performance, including grip strength. The elderly should be encouraged to pay attention to their oral health status.

On the other hand, in a South Korean study, while associations between the number of teeth and grip strength disappeared entirely in women after adjusting for socioeconomic status, use of dentures, health behavioral factors and general health, statistically significant connections remained in men^16^. Hamalainen et al. also found a positive correlation between the number of teeth and grip strength in men over the age of 80, but found no correlation in women^19^. A recent study similarly reported an association between tooth loss and mortality in older men, but not in women^39^. These findings are consistent with the results of our present study.

The differences in associations between men and women are difficult to explain. Variabilities in socioeconomic factors and social activity may be important influences on differences between men and women, and these factors may explain the lack of association between tooth loss and grip strength in women. In addition, reported results are not directly comparable, since there were differences in age distribution, methods of measuring grip strength, and adjustment for confounding factors in the previous studies^16,19,20,37^. Therefore, gender differences in the relationship between the number of teeth and grip strength is intriguing and deserves further study.

### The relationship between tooth loss and walking speed

Walking speed is a clinically useful indicator of mobility and overall health status in older adults and an independent predictor of adverse outcomes such as disability and mortality^40,41^. Decreases in walking speed likely result from a variety of factors that occur throughout life. Previous studies have confirmed that tooth loss is associated with slower average walking speed^42^. In addition, circulation levels of biomarkers of inflammation have been shown to be negatively associated with walking speed^43^. The mechanism of association can be explained in that an elevated level of proinflammatory cytokines in the serum of patients with periodontal disease may change the metabolism within local muscles groups leading to a decline in physical performance^44^. Sasajima et al.^45^ suggest that maintaining and activating oral function, including teeth retention and the ability to move tongue and lips, is associated with improved physical performance, including walking ability. These findings may suggest that imporoved oral care would help the elderly maintain a high quality of life and the ability to take care of daily. Accordingly, a recent cohort study showed that tooth loss was associated with an accelerated decline in walking speed in older adults and that inflammation may play a role in the association between tooth loss and walking speed decline^17^. However, the population included in this study had relatively high level of education, and the results may not be applicable to older suburban-dwelling adults with lower literacy rates.

Our study found that even after adjustment for confounding variables, tooth loss was associated with walking speed in women, but not in men. Our results are similar to a study from Japan^20^. Men, therefore, seem to be less prone to age-related bone and muscle decline than are postmenopausal women. While sex steroids can stimulate peak bone and muscle mass growth in adolescence and middle age and can prevent subsequent loss in older men, they have no effect on postmenopausal women^46^. Women are particularly vulnerable to decreased walking speed because of the dramatic decline in estrogen levels after menopause^47^. Men, conversely, tend to have enough muscle in their lower limbs to overcome the high rate of decrease of muscle mass with age^20^. These factors may explain our result that tooth loss was significantly related to walking speed in women, but not in men. Preventing tooth loss, then, may be more important for women.

### Limitations and strengths

Some limitations of this study are worth mentioning. First, measurement of oral health status was based on self-reports, not clinical examination. However, the validity and reliability of self-reported oral health has been established by multiple studies, and this method is widely used in epidemiological surveys. Second, this study is a cross-sectional study, and it was therefore impossible to establish causal relationship between tooth loss and sarcopenia and its components. We will explore its causality in a future longitudinal study. Third, our study only counted the number of tooth loss, but did not count the location, time or reason of loss of each tooth. Therefore, the interpretation of the results should be more cautious. Fourth, although the AWGS believes that the bioelectrical impedance analyzer can be used for muscle mass measurement, the accuracy of this tool for measuring muscle mass is controversial^48^. Finally, some older adults with poor physical performance may have been excluded from the study because it was not convenient for them to leave home to participate in a physical examination. The participants in this study are therefore older adults with good activity levels, and this self-selection may have an impact on the research results.

This study also has many strengths. First, for older adults, this study demonstrates that reducing tooth loss can improve functioning and reduce the risks of associated with physical decline. This result informs us that caregivers or individuals can prevent or reduce physical performance decline by improving oral health. Second, the study provided additional data regarding the oral health of a suburban community-dwelling population, and it therefore fills an important gap in understanding.

In conclusion, our research found that tooth loss is not related to sarcopenia and muscle mass, but tooth loss is related to physical performance and muscle strength. These associations and existing research evidence provide the possibility of improving the physical function of the older adults by improving oral health. The factors identified in this study can be used in suburban community health plans to maintain the physical function of older adults.

## Methods

### Participants

Our study population included residents from Hangu District, Tianjin and Chongming District, Shanghai, China. Participants were selected from 1614 individuals of age 60 years or above in these areas who joined the national free physical examination program conducted from August 2018 and June to July 2019. Participants with the following conditions were excluded from the study: (1) inability to communicate with interviewers or to grant informed consent (n = 21); (2) inability to perform the handgrip strength test or the 4 m walking test (n = 18); (3) failure to complete sarcopenia measurement (n = 36); or (4) failure to complete the questionnaire about oral care (n = 45). A total of 120 participants were excluded, leaving 1494 available for analysis.

### Assessment of height and weight

Subject height and weight were measured by personnel administering the survey after formal training by professionals. The ruler used to measure the height had a length of 200 centimeter and an precision of 0.1 cm and was pasted on a smooth and vertical wall prior to measurement. The measuring personnel determined the height of the subject as read and record the intersection reading between the highest point of the head and the vertical line of the column of the ruler. The weight of the subjects was measured by weighing scale with a weighing scale with an precision of 0.1 kg. During the measurement, the subjects should wear as little clothes and bare feet as possible.

### Assessment of the oral health status

Participants arriving at the community health service centers submitted a questionnaire via a face-to-face interview. The researchers assessed the condition of the participants' teeth by asking them how many teeth they had lost so far. Possible answers were none, 1, 2, from 3 to 5, from 6 to 9, and 10 or more teeth lost. Participants were then categorized into two groups based on the number of teeth missing: from 0 to 9 teeth lost or 10 or more teeth lost^49^. Besides, participants also self-reported denture status, answer "yes" or "no"; brushing frequency (" ≥ 2 times/day" or " < 2 times/day"); brushing time (" < 2 min" or " ≥ 2 min"); gingival bleeding ("yes" or "no").

### Assessment of sarcopenia

We used the definition from the Asian Working Group for Sarcopenia to assess sarcopenia^30^. Here, a person with low muscle mass, low muscle strength and/or low physical performance was identified as having sarcopenia. Low muscle mass was defined as a relative skeletal muscle mass index, which is a ratio of appendicular skeletal muscle mass (ASM) to height (Ht)^2^ of less than 7.0 kg/m^2^ in men and 5.7 kg/m^2^ in women. Low physical performance was defined as an average walking speed of less than or equal to 0.8 m/s. Low muscle strength was defined as a grip pressure of less than 26 kg for men and less than 18 kg for women.ASM values were obtained using a bioelectrical impedance analyzer (Inbody720; Bio space Co., Ltd, Seoul, Korea). Before measuring ASM, we measured it in strict accordance with the requirements of bioelectrical importance, and asked the patients to take off their socks before the test; Confirm the correct position of the contact point between hand, foot and electrode; Remove metal accessories from the body; It is forbidden to measure in patients with cardiac pacemaker.

### Assessment of muscle strength

Muscle strength was assessed via average grip strength of two attempts at maximum effort with a participant’s dominant hand. Grip strength was measured using a dynamometer (Grip-D; Takei Ltd, Niigata, Japan). The precision of this dynamometer is 0.1kg. The participants were standing during the test, the upper limb was abducted 45 degrees, and the elbow joint was straightened throughout the test. Participants were asked to make two attempts, one minute apart^50^. If the grip strength measured in the two attempts was very different, the participants were asked to rest for 15 minutes, and the average was reassessed.

### Assessment of physical performance

Walking speed was used as a marker of physical performance. To measure walking speed, we placed two photocells connected to a recording timer at the beginning and end of a 4 m course on site. Participants were asked to stand with their feet touching the starting line. When the tester gave the "start" command, participants began to walk at a normal speed, and the time between the activation of the first and second photocells was recorded^51^. The task was performed twice, and the average of the two times was considered to be the final walking speed. The precision of measurement time is 0.001 s.

### Assessment of covariates

Sociodemographic and behavioral characteristics and medical conditions were obtained from the participants through face-to-face consultations and questionnaires. Sociodemographic variables, including age, gender, marital status and educational level were assessed. Weight and height were measured, and BMI (kg/m^2^) was calculated. Marital status was categorized as widowed or not. Living conditions were divided into living alone or not.

Specifically, the "illiteracy" level means 0 years of formal education, "primary school" means between 0 and 7 years of formal education, and "middle school and above" means at least 7 year of formal education.  Behavioral characteristics included information on smoking (never, former smoker, or current smoker) and drinking (never, former drinker, occasional drinker or daily drinker) habits and were obtained from the questionnaire. Monthly income was also included, and categories were less than 157.2 USD, 157.2 to 471.6 USD, 471.6 to 786 USD, and greater than 786 USD. The presence of specific medical conditions, including diabetes, hypertension, dyslipidemia, were established using standardized criteria that combined information from history of physical illness evaluated on the basis of participants’ responses to questions, physician diagnoses, and statuses of corresponding treatments, pharmacological or otherwise. IPAQ was used to evaluate physical activity^52^. The Geriatric Depression Scale (GDS) was used to assess depressive symptoms in older adults; a GDS score of at least 11 was defined as depression^53^. We assessed nutritional status with Mini Nutritional Assessment (MNA) scores^54^.

### Statistical analyses

We separated the participants by sex and classified the number of teeth lost into two groups (0 to 9 and at least 10). Analysis of variance (ANOVA) and chi-square tests were conducted to compare the characteristics and lifestyle habits of individuals between the two groups. Binary logistic regression models and multivariate linear regression models were used for modelling the relationships among the three basic diagnostic components of sarcopenia and the number of teeth lost. Model 1 controlled for the covariates age, BMI and district; Model 2 was based on model 1, with additional adjustments of IPAQ, income, education, smoking, drinking and denture status; Model 3 was based on model 2, with additional adjustments of depression, nutrition status, hypertension and diabetes. Bilateral *p* < 0.05 was considered to be statistically significant. All statistical analyses were performed using IBM SPSS Statistics 25.0.

### Ethics approval

This study was approved by the Shanghai University of Medicine and Health Sciences ethics committee (2019-WJWXW-04-310108196508064467). All participants signed an informed consent form to participate before data collection. We confirm that all methods were performed in accordance with the relevant guidelines and regulations.
